# The Association between Adult Participation and the Engagement of Preschoolers with ASD

**DOI:** 10.1155/2016/6029837

**Published:** 2016-02-24

**Authors:** Ann M. Sam, Stephanie S. Reszka, Brian A. Boyd, Yi Pan, Kara Hume, Samuel L. Odom

**Affiliations:** ^1^Frank Porter Graham Child Development Institute, University of North Carolina, Chapel Hill, 517 S. Greensboro Road, Carrboro, NC 27510, USA; ^2^Department of Allied Health, University of North Carolina, Chapel Hill, NC 27599, USA

## Abstract

The ability for a child to engage in the classroom is associated with better academic outcomes. Yet, there is limited information on how child characteristics of autism and adult behavior impact engagement. This study examined (1) the pattern of adult participation and child engagement in preschool classrooms that serve children with ASD, (2) the associations between child engagement and adult participation, and (3) how characteristics of ASD (autism severity, language ability, and challenging behavior) moderate the relationship between adult participation and child engagement. Overall, children were less likely to be engaged when adults were actively or passively participating with them. Moderators impacted this relationship. Children with higher levels of autism severity were more likely to be engaged when adults were actively or passively participating with them. Similarly, children with lower language abilities were more likely to be engaged when adults were actively or passively participating with them. Finally, children with higher levels of challenging behaviors were less likely to be engaged when adults were actively or passively participating with them. These findings have important implications for how adults can best support the engagement of children with ASD.

## 1. Introduction 

The National Research Council of the United States [1] recommended that young children with autism spectrum disorder (ASD) receive intensive services for 25 hours per week to promote communication, social skills, cognitive skills, and appropriate behavior. While young children with ASD are spending many hours in various programs and classrooms, there is limited information concerning the efficacy of various comprehensive treatment models or how these models are implemented in schools [2]. Even less is known about how adults are participating with children in school-based classrooms and how adult participation impacts child engagement.

Teachers and other adults in classrooms play a key role in a child's developmental process as they assist in regulating a child's activity level and interactions with peers and adults [3]. Early interactions with teachers provide a working model of teacher-child relationships and create a pattern of how children engage with other adults in the school environment [3, 4]. The relationship between teachers and children can shape and alter developmental trajectories of children with more positive relationships with teachers associated with better academic and social outcomes for young children [5–7]. On a day to day basis, these interactions may influence the quality of instruction a child receives by impacting the level of a child's engagement in the classroom environment [7].

Child engagement is defined as “the amount of time children spend interacting with the environment (with adults, children, or other materials) in a manner that is developmentally appropriate” [8, p. 60]. Higher levels of engagement are correlated with better outcomes for learners [9, 10]. For example, kindergarteners who were identified as more engaged in classroom activities had higher literacy achievement scores at the end of the year than kindergarteners with lower levels of engagement in classroom activities [10].

The amount and quality of child engagement differ between children who are typically developing and children with disabilities. Kemp and colleagues [11] found that young children with a range of disabilities in inclusive childcare settings were engaged approximately for 67% of the time in free play, group, and meal activities. Brown and colleagues [12] found that preschool-aged children with disabilities in inclusive classrooms were engaged for 54% of the time. This level of engagement did not cause concern for Brown and colleagues [12] because the active engagement category did not take into account more passive forms of engagement such as listening to peers/teachers or transitioning between activities that the coding system might not have captured.

Odom and colleagues [13] found that the engagement level for children with ASD in inclusive settings (51%) was comparable to children with other disabilities (52%) and slightly lower when compared to children who were developing typically (59%). However, Kemp and colleagues [11] found that children with ASD were engaged during free play activities only for 47.6% of the time compared to children with other disabilities who were engaged in free play activities for 84.6% of the time in inclusive settings. Wong and Kasari [14] found that preschoolers with ASD in self-contained classrooms were unengaged for a lower amount of time (37%) during classroom activities (e.g., free play, centers, circle, and self-care activities). Possible reasons for the variability of engagement across research studies include various definitions of engagement used, observation of different activities within the preschool day (e.g., free play, circle time, and routines), type of classroom (e.g., self-contained versus inclusive), and child to adult ratio.

Child engagement levels can vary based upon specific child characteristics. For example, McWilliam and Bailey Jr. [15] observed that children with disabilities with younger developmental ages were more likely to be unengaged for longer period of time than typically developing children in an inclusive childcare center. A study by de Kruif and McWilliam [16] found that children identified by both teacher report and researchers' observations to be more developmentally mature (in an inclusive childcare center) spent more time in higher level engagement activities. Negative behavior exhibited by children has been shown to reduce the level of engagement [13]. Additionally, when children are not engaged, repetitive behaviors are more likely [16].

Adults in classrooms also play a key role in fostering child engagement. Adult participation has been defined in various ways throughout the literature [17–19]. Three common categories of adult participation are (1) active adult participation (i.e., interacting directly with the focal child), (2) passive adult participation (i.e., within a close distance to the focal child but not interacting directly), and (3) no adult participation towards a focal child (i.e., the adult is not interacting directly with the focal child or within close proximity of focal child). Powell and colleagues [18] found that adults in public school early childhood classrooms were out of range or disengaged with typically developing children in play settings for about 76% of the time.

The level of adult participation influences the degree of child engagement. For example, preschool children with disabilities demonstrated higher levels of engagement in activities when activities were selected by children versus adults [13]. When adults initiated the activity, children with disabilities interacted more often with adults rather than with other children [19]. Similarly, two-year-old boys with ASD were found to have higher levels of engagement when the child selected a toy to play with versus when a teacher made the toy selection for them [20]. Furthermore, McWilliam and colleagues [17] found that teacher's interaction style impacted the engagement of the preschoolers in an inclusive childcare setting. Specifically, when a teacher elaborated on a child's activity and provided the child with information, the child was more engaged than when the teacher responded to a child, asked a question of a child, or made a request of the child.

Researchers have found that teachers and adults interact with a child differently based upon disability status, thereby influencing child engagement levels. Adults tend to provide much more support and assistance to children with disabilities compared to children developing typically [12, 13]. Hamilton [21] found that teachers in inclusive classrooms focused more upon children with disabilities engaging with materials rather than interacting with peers. Overall, teachers and other adults tend to provide more support for children with disabilities to remain engaged when compared to peers developing typically.

While researchers have explored how children with and without disabilities engage in preschool classrooms throughout the day [11–13], very little is known about how preschoolers with ASD engage in free play or center time activities. Child characteristics, such as disability, maturity, and autism severity, have been shown to influence how children engage in classroom activities [11, 15, 16]. Yet, no studies have addressed how levels of adult participation (i.e., active adult participation, passive adult participation, or no adult participation) influence the engagement of children with ASD. Further, characteristics of ASD (e.g., severity, language ability, and challenging behavior) have not been examined to determine if these characteristics moderate the relationship between child engagement and adult participation.

Given the intensive education recommendations for preschool children with ASD [1], research needs to specifically focus on the relationships and interactions between adults in these programs and preschool children with ASD. Furthermore, research needs to address how specific characteristics of ASD (i.e., severity, language ability, and challenging behavior) moderate the relationship between adult participation and child engagement. The purpose of this study is to address the research gap through the following research questions:

- What are the patterns of adult participation and child engagement in preschool classrooms that serve children with ASD?
- What are the associations between child engagement and adult participation?
- Does autism severity moderate the relationship between adult participation and child engagement?
- Does language ability moderate the relationship between adult participation and child engagement?
- Does challenging behavior moderate the relationship between adult participation and child engagement?

## 2. Method

This study was part of a larger study comparing two comprehensive treatment models, high fidelity LEAP (i.e., Learning Experiences Alternative Programs for Preschoolers and Parents) and TEACCH Autism Program classrooms, to each other and a control condition (Business as Usual (BAU)). The term BAU was used instead of treatment as usual to refer to non-model-specific special education programs where children receive standard or recommended school-based services [22]. While the current study did not address how engagement of children or participation of adults varied as a function of the model, model type was used as a covariate to remove possible confounds (see [22]). Sites included four states: North Carolina, Florida, Colorado, and Minnesota. For the larger study, data were collected at three time points: pretest (start of school year), posttest (end of school year), and follow-up (6 months after posttest collected). For this study, only data from the first time point were used.

### 2.1. Program Settings

Established in 1972 by Schopler, the TEACCH Autism Program foundation is in behavioral principles and cognitive social learning theory. The TEACCH program stresses using the environment to enhance the learning of individuals with ASD [23]. In 1981, Strain established LEAP, a preschool program which includes children with ASD in an inclusive program. LEAP adapts early childhood curriculum and applied behavioral analysis and developmental theory [24–26]. While BAU classrooms include children with ASD, the curriculum used is not designed to specifically address characteristics of ASD. These programs use an eclectic approach to address the needs of children with ASD [27].

### 2.2. Participants

Classrooms met the following inclusion/exclusion criteria: (1) classrooms were within a public school system, (2) teacher was licensed to teach in respective state, (3) TEACCH and LEAP teachers attended a formal training either from personnel directly associated with the model/program or from someone formally trained (trainings varied from 3 to 5 days and occurred with no affiliation with the study), (4) teachers worked in their respective classroom type for a minimum of two years prior to the start of the study, and (5) teachers met prior-determined criteria of an “average” rating (score of 3 out 5) on four subscales of a classroom quality measure, the PDA Program Assessment [28].

To be enrolled, children met the following criteria: (1) between 3 and 5 years of age at time of enrollment; (2) previous clinical diagnosis or educational label consistent with ASD or developmental delay; (3) meeting diagnostic criteria on Autism Diagnostic Observation Schedule (ADOS) [29]; and (4) no previous exposure to the comparison treatment model (e.g., a child enrolled in a TEACCH classroom could not have prior exposure to the LEAP model). Children were excluded from the study with significant uncorrected vision or hearing impairment, uncontrolled seizure disorder, or traumatic brain injury. Lastly, in order to complete parent rating scales, families had to be proficient in English.

Child participants included 187 preschool-aged children (age 3–5) diagnosed with ASD. Children were enrolled in TEACCH programs (43.16%), BAU classrooms (28.95%), and LEAP classrooms (27.89%). Child participants included 83.96% males. Child participants were white (79.86%), black (10.70%), Asian (5.35%), multiracial (3.74%), and other (0.53%). At pretest, children ranged in age from 2.9 to 5.18 years (mean 4.01 years).

Teacher participants included 69 female teachers and 1 male teacher. Teachers were white (97.14%) and black (2.86%). The mean number of years teaching was 10.37 years (range 2–29.5 years). Teachers had a master's degree (55.7%), a bachelor's degree (38.6%), education above a master's degree (4.3%), or an associate's degree (1.43%). Within classrooms, the mean child to adult ratio was 3.55 students per teacher.

### 2.3. Measures

Child assessments were conducted by trained research staff within a six-week period at the beginning of the school year. See Table 1 for assessment information. The Mullen Standard Score at pretest was 64.12 (SD 18.99) [30]. The Preschool Language Scale-IV (PLS-IV) assesses auditory comprehension and expressive communication to obtain a total language score [31]. The mean score for the PLS-IV was 63.96. The Childhood Autism Rating Scale (CARS) assesses the severity of autism as normal (scores below 30), mildly and moderately autistic (scores 30–36.5), and severely autistic (scores 37–60) [32]. The mean CARS score was 33.43. The Child Behavior Checklist (CBCL) provides a summary of internalizing, externalizing, and total problems [33]. The total problems raw score was used to determine challenging behavior. The mean CBCL total problem raw score was 51.23 (normative sample mean is 33.30 with a standard deviation of 18.70).

Research staff videotaped each participant for a total of 30 minutes during center time (a common feature across all classrooms where children participate in a variety of activities located in different centers such as blocks or art). During center time, children selected different activity areas (including dramatic play, large blocks, art, computer, sensory, and manipulatives). PROCODER software [34] was used to assist in coding each video using the* Code for Active Student Participation and Engagement-Revised (CASPER-III)* [35].* CASPER-III* is an ecobehavioral assessment. Ecobehavioral assessments have been used to examine such variables as child engagement, teacher instruction/support, and peer social interactions [12, 21, 36–38]. These assessments typically examine three variables: adult behavior, classroom/environment characteristics, and student behavior [36]. The* CASPER-III* variables included Activity Area, Group Arrangement, Child Behavior, Initiator of Activity, Adult Support, and Social Behavior [35]. Momentary time sampling at 10-second intervals was used to code each video.

Videos were coded by one of our trained research assistants trained with the* CASPER-III Training Manual for Observers* [35]. Raters practiced coding until all raters reached consensus with at least 80% agreement (i.e., the number of agreements divided by the number of agreements plus disagreements) or a Kappa of at least 0.80 for each variable. Twenty percent of observations were coded by an additional rater for interobserver agreement for each variable (Group Arrangement, Adult Support, and Child Behavior). Both* Kappa* and agreement measures were used. Note that the agreement measure is based upon observed agreement. See Table 2 for the interobserver agreement.

The current study examined both adult participation in reference to an identified focal child and the focal child's engagement level. Each child participant served as a focal child. The focal child's environmental context was videotaped (i.e., the identified focal child, the center or area the focal child participated in, and other children/adults in the immediate area were filmed). The child engagement and adult participation variables were created by combining existing* CASPER* variables. Adult participation included the* CASPER* variables of Active Participation, Passive Participation, and No Participation.  Table 3 presents the* CASPER* variables and definitions used to create these variables. Child engagement variables included Active Child Engagement and No Active Child Engagement by combining* CASPER* variables.  Table 3 presents the* CASPER* variables and definitions used to create the child engagement variables.

### 2.4. Data Analysis

Descriptive statistics were used to determine the pattern of child engagement and adult participation. To address the repeated measures within children, children nested within classrooms, and both adult level and child level variables, multilevel logistic regression was used for the remaining research questions. The question regarding the associations between child engagement and active (*γ*_001_  adultactive_*tij*_) and passive (*γ*_002_  adultpassive_*tij*_) adult participation was addressed using multilevel logistic regression controlling for comprehensive model type (TEACCH (*γ*_101_  TEACCH_*j*_) and LEAP (*γ*_102_  LEAP_*j*_)), child to adult ratio (*γ*_100_  ratio_*j*_), child ASD severity (*γ*_010_  severity_*ij*_), child language ability (*γ*_011_  language_*ij*_), and child challenging behavior (*γ*_012_  behavior_*ij*_) (Model 1). Model 1 was

(1) logit Ptij=γ000+γ001 adultactivetij+γ002 adultpassivetij+γ100 ratioj+γ101 TEACCHj+γ102 LEAPj+γ010 severityij+γ011 languageij+γ012 behaviorij+μoij+R1j.

Finally, to answer the three moderation effect questions, one interaction model was used. Covariates included comprehensive model type (TEACCH (*γ*_101_  TEACCH_*j*_) and LEAP (*γ*_102_  LEAP_*j*_)), child to adult ratio (*γ*_100_  ratio_*j*_), child ASD severity (*γ*_010_  severity_*ij*_), child language ability (*γ*_011_  language_*ij*_), child challenging behavior (*γ*_012_  behavior_*ij*_), interactions between active adult participation (*γ*_001_  adultactive_*tij*_) and each of the three moderators (child ASD severity (*γ*_003_  adultactive_*tij*_ *∗*severity_*ij*_), child language ability (*γ*_003_  adultactive_*tij*_ *∗*language_*ij*_), child behavioral issue (*γ*_003_adultactive_*tij*_ *∗*behavior_*ij*_)) and interactions between passive adult participation (*γ*_002_  adultpassive_*tij*_) and each of the three moderators (child ASD severity (*γ*_004_  adultpassive_*tij*_ *∗*severity_*ij*_), child language ability (*γ*_004_  adultpassive_*tij*_ *∗*language_*ij*_), and child behavioral issue (*γ*_004_  adultpassive_*tij*_ *∗*behavior_*ij*_)) (Model 2). Model 2 was

(2) logit Ptij=γ000+γ001 adultactivetij+γ002 adultpassivetij+γ003 adultactivetij∗severityij+γ004 adultpassivetij∗severityij+γ003 adultactivetij∗languageij+γ004 adultpassivetij∗languageij+γ003 adultactivetij∗behaviorij+γ004 adultpassivetij∗behaviorij+γ100 ratioj+γ101 TEACCHj+γ102 LEAPj+γ010 severityij+γ011 languageij+γ012 behaviorij+μoij+R1j.

Post hoc tests were used to examine the linear combinations of parameter estimates at crucial values (i.e., at the mean, a standard deviation above the mean, and a standard deviation below the mean) for significant associations.

## 3. Results 

The first research question examined the pattern of child engagement and adult participation. Adults spent most of their time passively participating with focal children (36.5%), followed by actively participating with focal children (34.1%), and then no adult participation with focal children (29.4%). Children spent the majority of their time engaged in activities (72.3%) with only 27.7% of time spent not engaged.

Associations between child engagement and adult participation were examined in the second research questions. Model 1 results are presented in Table 4. Focal children were less likely to be engaged when adults were actively participating with a focal child (*t *= −2.52, *p* = 0.012, log OR = −0.108, and SE = 0.043). The odds ratio (OR) of focal child engagement between actively and not actively participating adults was estimated at 0.90. Focal children were also less likely to be engaged when adults were passively participating with a focal child (*t* = −2.25, *p* = 0.024, log OR = −0.095, and SE = 0.042). The estimated OR was 0.91.

**Figure 1:**
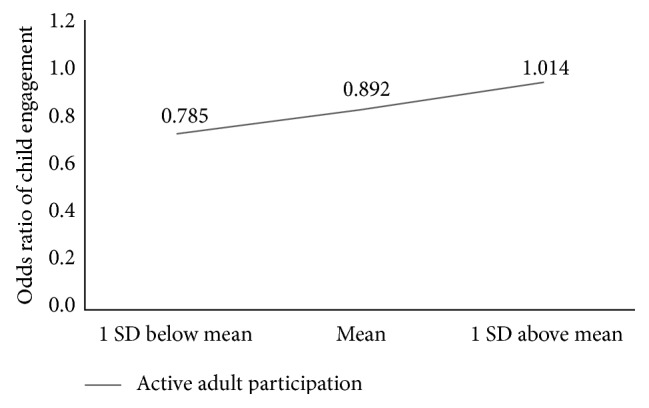
Interaction of active adult participation and child ASD severity on child engagement.

The final three research questions addressed how autism severity, child language ability, and child challenging behavior moderated the relationship between adult participation and child engagement. Model 2 results are presented in Table 5. The interaction between child engagement and active adult participation was moderated by autism severity (log OR = 0.128, SE = 0.057, *t* = 2.27, and *p* = 0.024) with active adult participation supporting child engagement. Children with higher autism severity ratings (i.e., CARS scores a standard deviation above the mean) were likely to be engaged when adults were actively participating. Post hoc test examined the linear combinations of parameter estimates at crucial values (i.e., at the mean and a standard deviation above and below the mean). The estimated OR of engagement between actively and not actively participating adults for children with CARS scores at one standard deviation above the mean (+1 SD) was not statistically significantly different from 1 (log OR = 0.014, SE = 0.074,* t* = 0.19, and *p* = 0.8493); the estimated OR for children with CARS scores at the mean was 0.89 (log OR = −0.114, SE = 0.043,* t* = −2.63, and *p* = 0.0085); the estimated OR for children with CARS one standard deviation below the mean (−1 SD) was 0.78 (log OR = −0.243, SE = 0.068,* t* = −3.54, and *p* = 0.0004).  Figure 1 shows the odds ratios of child engagement between active adult participation and no active adult participation for children with different levels of ASD severity measured by CARS. No evidence was found for differential associations between child engagement and passive adult participation for children with different ASD severity levels (log OR = 0.034, SE = 0.056, *t* = 0.6, and *p* = 0.549). Overall, children with higher autism severity ratings were more likely to be involved when adults were interacting with them.

**Figure 2:**
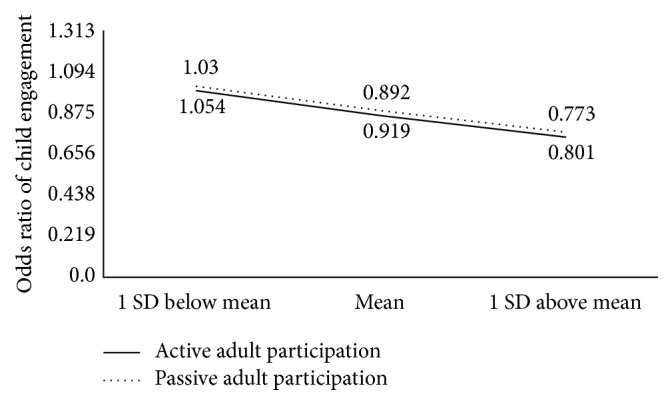
Interaction of active/passive adult participation and child language ability on child engagement.

As shown in Table 5, there was evidence that the association between child engagement and active adult participation was negatively moderated by child language ability (log OR = −0.143, SE = 0.059,* t* = −2.43, and *p* = 0.015). Post hoc analysis showed that estimated OR of engagement between actively and not actively participating adults for children with language scores at +1 SD was 0.77 (log OR = −0.258, SE = 0.076,* t* = −3.37, and *p* = 0.0008); the estimated OR for children with language ability at the mean was 0.89 (log OR = −0.114, SE = 0.043,* t* = −2.63, and *p* = 0.0085); the estimated OR for children with language ability at −1 SD was not statistically significantly different from 1 (log OR = 0.029, SE = 0.070, *t* = 0.42, and *p* = 0.6768). The association between passive adult participation and child engagement was also negatively moderated by child language ability (log OR = −0.137, SE = 0.057,* t* = −2.39, and *p* = 0.0169). Post hoc test showed that estimated OR of engagement between passively and not passively participating adults for children with language score at +1 SD was 0.80 (log OR = −0.221, SE = 0.074,* t* = −3, and *p* = 0.0027); the estimated OR for students with language score at the mean was 0.92 (log OR = −0.084, SE = 0.042,* t* = −1.99, and *p* = 0.047); the estimated OR for students with language score at −1 SD was not statistically significantly different from 1 (log OR = 0.053, SE = 0.069,* t* = 0.76, and *p* = 0.4445).  Figure 2 shows the impact of the significant interaction between active and passive adult participation and child language ability on child engagement. Overall, children with higher language abilities were less likely to be engaged when adults were actively or passively participating with them.

**Figure 3:**
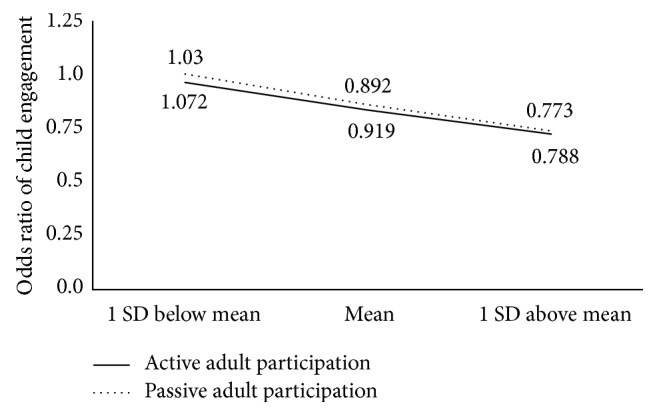
Interaction of active/passive adult participation and Child Behavior on child engagement.

Similar to the effect of language scores, the association between child engagement and active adult participation was negatively moderated by child challenging behavior (log OR = −0.143, SE = 0.050,* t* = −2.88, *p* = 0.004, and *p* = 0.0039). Post hoc analysis showed that estimated OR of engagement between actively and not actively participating adults for children with problem behavior score at +1 SD was 0.77 (log OR = −0.258, SE = 0.065,* t* = −3.96, and *p* = 0.0001); the estimated OR for children with problem behavior score at the mean was 0.89 (log OR = −0.114, SE = 0.043,* t* = −2.63, and *p* = 0.0085); the estimated OR for children with problem behavior score at −1 SD was not statistically significantly different from 1 (log OR = 0.029, SE = 0.067, *t* = 0.43, and *p* = 0.6638). Similarly, the association between passive adult participation and child engagement was negatively moderated by child challenging behavior (log OR = −0.154, SE = 0.049,* t* = −3.15, and *p* = 0.002). Post hoc test showed that estimated OR of engagement between passively and not passively participating adults for children with problem behavior score at +1 SD was 0.79 (log OR = −0.238, SE = 0.064,* t* = −3.71, and *p* = 0.002); the estimated OR for students with language score at the mean was 0.92 (log OR = −0.084, SE = 0.042,* t* = −1.99, and *p* = 0.047); the estimated OR for students with language score at −1 SD was not statistically significantly different from 1 (log OR = 0.069, SE = 0.065,* t* = 1.06, and *p* = 0.2873).  Figure 3 shows the impact of the significant interactions between active and passive adult participation and child challenging behavior on child engagement. Overall, children rated with higher amounts of challenging behavior were less likely to be engaged when adults were actively or passively participating with them.

To summarize, higher levels of adult participation were associated with less child engagement. However, when children had greater autism severity or less language ability, higher levels of adult participation were associated with higher levels of child engagement. For children with more challenging behaviors, higher adult participation was associated with less engagement.

## 4. Discussion

This study provides information on how adult participation is associated with child engagement in preschool classrooms that serve children with ASD. This is the first study that explored how specific characteristics (i.e., autism severity, child language ability, and challenging behavior) moderate this relationship.

During center time activities, preschoolers with ASD were engaged in classroom activities for 72.3% of the time. The percentage of time engaged was higher in this study than previous research which found that children with ASD were engaged approximately half the time (47.6% and 51%) [11, 13]. Brown and colleagues [12] observed preschoolers with disabilities in inclusive settings across multiple activities and settings resulting in numerous transitions where students might not be “engaged.” In contrast, this study focused on center time and included both inclusive and separate/self-contained settings. While preschoolers could transition between activities, the transitions were minimal and relatively short in duration. The inclusion of high quality classrooms in the current study is another possible explanation for the higher rates of child engagement. Previous studies have linked classroom quality to child engagement levels in preschool, kindergarten, and third grade classrooms [9, 10, 15]. While the models used for analysis controlled for covariates of model type (LEAP an inclusive setting and TEACCH a self-contained setting) and adult to child ratios, the teachers in these classrooms had received formal trainings on models and classrooms met quality measures (see [23]).

Adults divided their time with focal children fairly evenly spending 36.5% of the time passively participating with the identified focal child, 34.1% of the time actively participating, and 29.4% of the time not participating with the focal child. Adults in this study spent more time actively and passively participating with focal children than what is found in a previous study for preschoolers without disabilities in urban public preschool classrooms [18]. The current studies definition of adult participation and inclusion of high quality classrooms could account for these differences. The inclusion of high quality classrooms possibly resulted in preselecting adults who were more likely to regularly interact with children.

The current study found that children were less likely to be engaged when an adult was actively or passively participating with them. To explore this relationship further, this study examined the possible impact of several potential moderators on child engagement. First, autism severity positively moderated the relationship between adult participation and child engagement with children rated with more severe forms of autism more likely to be engaged when adults were actively participating with them. Previous research found that adults are more likely to interact with children who are involved in more solitary activities or passive forms of play in childcare centers and inclusive preschools [39, 40]. Given this, adults might seek out children with more severe forms of ASD who are isolated and need more support and guidance in the preschool classroom. Second, the association between child engagement and adult participation was negatively moderated by child language ability with children with lower language abilities more likely to be engaged when adults were actively or passively participating with them. Similar to children with more severe forms of autism, children with lower language abilities may have required more assistance from adults in the classroom to engage in classroom activities successfully. Finally, challenging behavior was negatively associated with adult participation and child engagement. Children with higher amounts of challenging behavior engaged less frequently when adults were actively or passively participating with them. This finding is supported by previous research that found that children with more challenging behavior are less likely to be engaged in activities in inclusive classrooms [13]. Children with more challenging behavior might require additional support from adults in the classroom. For example, when a child is exhibiting challenging behavior (such as throwing toys, hitting, and kicking) an adult is more likely to support the child to ensure the safety of the child and other peers in the class. However, the behavior of the child during this period would be coded as not engaged.

Several limitations should be noted for this study. First, the analysis only included data collected at pretest during the beginning of the year. Adults might alter their behavior towards children throughout the year as children gain more independence. Next, the quality of the video impacted the ability to code behaviors. For example, the context of the video was not always captured. If an adult or child was off screen the time point, coding was not possible during the affected time point. Third, the definition of child engagement was based upon an ecobehavioral assessment and differs from other engagement literatures [12]. A more precise coding of child engagement using hierarchical engagement codes might have resulted in different findings. The cooccurrence of adult participation and child engagement was examined which prevented the ability to determine how adult participation predicts child engagement. Finally, past research regarding child engagement levels has focused on inclusive settings [11, 13] or self-contained, separate settings [14]. This study included both self-contained and inclusive classroom settings. While treatment model (i.e., TEACCH and LEAP) was controlled for in the analysis, the results were not interpreted by inclusive or separate settings.

## 5. Conclusion

The present study provides information on the association of adult behavior and the engagement of preschoolers with ASD. Given that children were more engaged when adults were not present or interacting with the focal child, arranging the environment to promote the active engagement of children is critical. Arranging the environment to promote learning has been emphasized as best practice for young children [41] and as helpful for children with ASD [24]. The arrangement of the environment might be particularly important for children who display more challenging behaviors as these children were less likely to be engaged when adults were actively participating with them. Children with more severe forms of autism and less language ability were more likely to be actively engaged when adults were actively participating with them. These children might need more support from adults to engage with materials or the environment. The current study examined the association between child engagement and adult participation based upon the selected characteristics as moderators. However, these moderators are not independent of one another. For example, a child with ASD might display challenging behavior, low language ability, and more severe forms of ASD. This child might benefit from a more structured environment and support from an adult when needed to actively engage with the environment.

Future research is needed to determine how potential characteristics of adults (e.g., education level, experience, training, and burnout) moderate child outcomes. Additionally, future research should focus on exploring the causal relationship between adult behavior and child engagement. Engagement is critical to enhance positive outcomes for children. Based upon this study, child moderators have the potential to impact how teachers and other adults can best support the engagement of children.

## Figures and Tables

**Table 1:** Child assessments.

Assessment	Mean	SD	Range
Mullen (standard score)	64.12	18.99	49–136
CARS	33.43	7.26	15.5–55.5
PLS4	63.96	28.17	4–129
CBCL	51.23	22.92	3–107

**Table 2:** Interobserver agreement.

	*A*/(*A* + *D*)		Kappa	
	Mean	Range	Mean	Range
Adult Support	0.93	0.73–0.99	0.84	0.48–0.94
Arrangement	0.93	0.83–0.99	0.88	0.70–0.99
Child Behavior	0.9	0.73–0.97	0.83	0.57–0.91

**Table 3:** Operational definitions.

	*CASPER* variables	*CASPER* definitions
Adult participation		
Active Participation	Direction Adult Support	Adult provides instruction to focal child or assistance in performing a task or activity.
	Adult approval	Adult expresses praise, appreciation, or satisfaction (verbally or physically) with the focal child.
	Adult comment	Adult talks or gestures to focal child without providing direct support or approval.
Passive Participation	Group discussion/directions	Adult reads aloud to a group of children, sings to group, or gives direction to a group of children including focal child.
	No adult behavior to focal child and one of the following group arrangements: 1 : 1 with adult Small group with adult Large group with adult	Adult is directing no coded behavior to focal child or group of children with focal child, but adult is in close proximity to focal child.
No Participation	No adult behavior to focal child and one of the following group arrangements: Solitary Small group Large group	Adult is directing no coded behavior to focal child or a group of children with focal child, and an adult is not in close proximity to focal child.
Child engagement		
Active Child Engagement	Book	Focal child actively involved with books.
	Preacademics	Focal child engages in preacademic behavior.
	Pretend/sociodramatic play	Focal child uses objects or materials in symbolic manner or performs a role in play theme.
	Art	Focal child interacting in art activity.
	Games with rules	Focal child participating in game with rules.
	Dance/music/recitation	Focal child engaging in songs, poems, nursery rhymes, or dances.
	Self-care/self-help	Focal child involved in caring for his or her personal needs.
	Manipulating	Focal child using coordinated eye-hand movements to interact meaningfully with materials or objects.
	Large motor	Focal child using large muscle movements.
	Clean-up	Focal child is putting away toys, equipment, furniture, dishes, and so forth.
No Active Child Engagement	Not engaged	Focal child is not actively engaged in any *CASPER* child engagement category.
	Stereotypic/repetitive	Focal child involved in some type of repetitive or stereotypic behavior.

**Table 4:** Model one results for associations between child engagement and adult participation.

Effect	Est.	SE	*t* stat.	*p*
Intercept	1.066	0.135	7.91	<0.0001
Active adult participation	−0.108	0.043	−2.52	0.0116
Passive adult participation	−0.095	0.042	−2.25	0.0243
TEACCH	0.031	0.190	0.16	0.8693
LEAP	0.164	0.182	0.9	0.3679
PLS 4	0.371	0.051	7.27	<0.0001
CARS	−0.021	0.044	−0.49	<0.6268
CBCL	−0.225	0.037	−6.01	<0.0001
Child-adult ratio	−0.159	0.081	−1.95	0.0513

**Table 5:** Model 2 results for moderators of relationship between child engagement and adult participation.

Effect	Est.	SE	*t* stat.	*p*
Intercept	1.055	0.136	7.74	<0.0001
Active adult participation	−0.114	0.043	−2.63	0.0085
Passive adult participation	−0.084	0.042	−1.99	0.047
TEACCH	0.030	0.192	0.15	0.8772
LEAP	0.161	0.184	0.88	0.3809
PLS 4	0.476	0.066	7.23	<0.0001
CARS	−0.090	0.061	−1.48	0.1385
CBCL	−0.120	0.049	−2.43	0.0152
Child-adult ratio	−0.167	0.082	−2.03	0.0423
Active adult participation *∗* PLS 4	−0.143	0.059	−2.43	0.0151
Passive adult participation *∗* PLS 4	−0.137	0.057	−2.39	0.0169
Active adult participation *∗* CARS	0.128	0.057	2.27	0.0235
Passive adult participation *∗* CARS	0.034	0.056	0.6	0.5488
Active adult participation *∗* CBCL	−0.143	0.050	−2.88	0.0039
Passive adult participation *∗* CBCL	−0.154	0.049	−3.15	0.0016
